# Health and Disease

**Published:** 1879-11

**Authors:** 


### HEALTH AND DISEASE.

**And so, although the thermometer was about twenty above zero, and a strong cold west wind was blowing, we went to work, and when it was completed, the whole body was in a profuse perspiration; but the neck, throat, and upper part of the chest felt as if they were frozen a foot, more or less, deep; and then came out the expression, with very considerable unction, "What an unmitigated fool have I been, to be work-**

ing all this time with head, and neck, and chest exposed to such a piercing cold wind!" Then came up visions of croup, diptheria, quinzy, putrid sore throat, and a dozen other hob-goblins, any one of which might have put us past cure within forty-eight hours; and already there was inability to draw a full, deep, satisfactory breath; and now, reader, for the idea which we wanted to impress on your mind, at the expense of being thought a very egoistic individual; but if we can put you in a way of saving your in—? valuable life! we will feel well compensated. The only course to pursue was to keep up a vigorous circulation, so as to prevent chilliness; for if that had taken place, pneumonia would have been a pretty certain event; but exercise for this purpose was out of the question, because we were already jaded out; perhaps a non-te-totaller would have resorted to a deep and hearty swig of wiskey; but that was not in our line, and besides there was not a drop in the house, then there remained but one means left, the application of heat, by means of fire and hot water; so we paddled the hands and face in water as hot as could be borne, and it was exceedingly grateful; then dressed as rapidly as possible and sat by a red-hot kitchen range, until the whole body was perspiring again; took breakfast, and went to writing an article for the Journal about murdering children in the public schools. As far as we know, we did not suffer the slightest injury by the unwise exposures which have just been detailed. Perhaps the first thought of the reader would have been to have applied the hot water to the neck, throat and upper part of the chest; that would have been inconvenient, would have been but partial, and would have dribbled the upper end of the flannel shirt, which was already wet with perspiration and was also very cold, but the hands and the face were at the extremities, and hot water would bring the blood to them, through the parts which seemed to be so cold, and thus warm them up in its progress; the hands could be kept continously hot, by keeping them immersed in hot water; the same with the face; but to apply water to the neck, with the hand or sponge, would be but for an instant, and only for the space which was covered by the hand, and it may be useful to remark here, that the safest way to cool off, even in summer, is to dip the hands in hot water and raise them in the air; do this successively; the philosophy of it is, that the evaporation of heat is very rapid, and is carried off by the steam caused by the hot water and warm skin; cold water will do the same thing, because the heat of the skin converts it into steam, but it is a harsh method, and is never so safe; and many times, when a person comes into the house from a cold walk or ride, feeling as if a chill were iminent, a most comfortable method of averting the chill, and of warming up the whole blood, is to immerse the hands in hot water, and keep them in motion; for if allowed to remain still, the layer of water next to them becomes in a measure cold; while by moving them about, the skin finds new layers of hot water, and thus every motion is grateful. The same may be said as to the feet; it is certainly a very agreeably method of getting warmed up; and when you have become so, it is well to dip both hands and feet into a vessel of cold water, for a second or two. Of course, if the extremities are frozen, stiff with cold, or without feeling, then snow or very cold water should be applied under the supervision of a physician.

But was it not undignified for us to be shovelling snow in trowserloons and shoes? we do not consider any thing "infra—dig" which promotes health and is a help to others. Trousseau, one of the greatest medical men of his age, whom kings and queens, within ten years, have been glad to have it in their power to consult, tells us that he brought on a very severe attack of asthma by concealing himself in the hayloft to see whether his coachman did not steal his horse feed.

The great Newton was found once in a less dignified employment, smoking a vulgar pipe, puffing away for life, like a young steam engine drawing smoke in and then as soon as it got it in, go about hustling it out and this for a whole hour at a time; and what's the use of it? it makes you no wiser and no better; you infume your whole clothing, you spit and hawk and skatter around your saliva, on carpet, floor furniture, and the dress of your friends; bespattering your own clothing with nauseous looking stains, and often having a streak of filthy slaver extending from the corners of the mouth, and than to find ourself such a helpless slave to the beastly custom ; but this is off the subject; Sir Isaac's sweetheart is reported to have been sitting by him, and wishing to adjust the fire of his pipe he took her finger, instead of his own, and so burned it, as to outrage her feelings, and she never would have any thing to do with him more; so we think our action in the premises will favorably compare with that of the great Physician or the greater Philosopher. But let us here tell a valuable secret as to the manner in which great men employ their time.

